# The Role of Interleukin-6 and C-Reactive Protein in Non-Thyroidal Illness in Premature Infants Followed in Neonatal Intensive Care Unit

**DOI:** 10.4274/jcrpe.625

**Published:** 2012-06-09

**Authors:** Dilek Dilli, Uğur Dilmen

**Affiliations:** 1 Zekai Tahir Burak Maternity Teaching Hospital, Department of Neonatology, Ankara, Turkey; +90 312 306 52 70dilekdilli2@yahoo.com

**Keywords:** Premature, newborn, Interleukin-6, C-reactive protein, thyroid hormone

## Abstract

**Objective:** To investigate the role of interleukin-6 (IL-6) and C-reactive protein (CRP) in non-thyroidal illness (NTI) in premature infants.

**Methods:** Serum levels of IL-6 and CRP, thyroid-stimulating hormone (TSH), total thyroxine (T4), free T4 (fT4), total triiodothyronine (T3), and free T3 (fT3) were determined at the 1st, 2nd and 4th weeks of postnatal life in 148 premature infants born before 33 weeks of gestation.

**Results:** At the 1st week, serum T3 was negatively correlated with IL-6(r= -0.33, p= 0.001) and CRP (r= -0.17, p= 0.03). Serum T3 was negatively and more strongly correlated with IL-6 (r= -0.49, p= 0.001) and CRP (r=- 0.33,p= 0.03) at the 2nd week, at which time sepsis frequency and low T3 rates were the highest. At the 4th week, mortality rate was higher among infants with lower T3 levels.

**Conclusions:** High IL-6 and CRP values related to neonatal sepsis might have a significant role in the pathogenesis of NTI in premature infants.

**Conflict of interest:**None declared.

## INTRODUCTION

Despite the absence of thyroid disease, patients with non-thyroidal illness (NTI) frequently have changes in serum levels of thyroid hormones that may suggest thyroid dysfunction (1). In such patients, the most consistent finding is a low serum total triiodothyronine (T3) level (<1 nmol/L) (2). This decrease is frequently accompanied with an elevated reverse T3 (rT3) level. Total thyroxine (T4) may be low or normal, and free T4 (fT4) may also be normal depending on the metabolic clearance rate of T4.

Serum T3 concentrations are reported to range from 160 to 240 ng/dL in 2-4-week-old healthy term infants (3), while mean ± SEM values for T3 in infants born between 23-29th weeks of gestation are reported as 0.8 ± 0.07nmol/L in the 2nd week of life (4). Therefore, thyroid tests in preterm infants may show a confusing situation when the infants are exposed to NTI.

Changes in thyroid functions occur in patients with a variety of NTI, as observed in those who are admitted to a medical intensive care unit (5,6,7,8,9). In preterm infants, respiratory distress syndrome (RDS) is the most frequently encountered cause of NTI (1,10,11).It was reported that cytokines have several effects on thyroid functions and can modulate the pituitary-thyroid axis (12,13,14). In this study, we aimed to evaluate the potential relation of serum interleukin-6 (IL-6) and C-reactive protein (CRP) with alterations in thyroid hormone levels seen in NTI. 

## METHODS

The study was conducted in the Ministry of Health Zekai Tahir Burak Maternity Teaching Hospital in Ankara, Turkey. The data were prospectively collected during the period between 1 June 2008 and 31 May 2009.

During the study period, a total of 497 premature infants with a gestational age of less than 33 weeks were admitted to the Neonatal Intensive Care Unit. The infants who died within the first week of life, those with disabling congenital malformations, those whose mothers had thyroid disorders and/or were on any thyroid medications, and infants of mothers who were unable to provide informed consent were excluded.

The subjects were evaluated at the end of their 1^st^, 2^nd^ and 4^th^ weeks of life.

At the end of their first week, 180 infants were available for the study. However, infants whose serum thyroid-stimulating hormone (TSH) levels were >30 mIU/L and those whose blood samples could not be obtained were excluded and data were available for 148, 127, and 80 infants at their postnatal 1^st^, 2^nd^, and 4^th^ weeks, respectively.

Infants with T3 < 1nmol/L (2) levels were accepted as having NTI. These infants were divided into two groups according to their T3 values in their respective age groups (1^st^, 2^nd^, and 4^th^ weeks of life). Thus, Group 1 consisted of infants with T3 levels <1nmol/L and Group 2 - of those with T3 levels ≥1nmol/L.

The subjects’ gestational age, birth weight, gender, and5-min Apgar score (< 6) were recorded from their medical files. Data pertaining to mechanical ventilatory support (>10 d), intracranial hemorrhage (ICH) (≥ grade II), RDS (with surfactant treatment), persistent ductus arteriosus (PDA) (with ibuprofen treatment), neonatal sepsis (clinical or proven) (17,18), necrotizing enterocolitis (NEC) (≥ grade II), bronchopulmonary dysplasia (BPD) (with need of oxygen support at postnatal 28 d), retinopathy of prematurity (ROP) (requiring laser photocoagulation), length of hospital stay, and mortality rate were prospectively recorded.

Blood samples were taken at the end of the 1^st^, 2^nd^, and 4^th^ weeks of life. Plasma levels of IL-6 (Siemens Diagnostic Product Corporation, Los Angeles, CA 90045-6900, USA) and serum concentrations of CRP (CRP latex HS, Roche kit, Roche Diagnostics, GmbH, D-68298 Mannheim, Germany) were measured. Thyroid function tests including T3, T4 (BioSource Europe S.A., Nivelles, Belgium), fT3, fT4, and TSH (Roche Diagnostics; Indianapolis, IN, USA) were performed. Blood cultures (Becton-Dickinson, Sparks, Maryland, USA) were done to define proven sepsis.

The study protocol was approved by the Local Ethics Committee. All parents were fully informed about the investigational nature of this study as well as its aim and provided written consent.

## STATISTICAL ANALYSIS

All data analyses were performed using the SPSS software (Statistical Package for the Social Sciences, version 17.0, SPSS Inc, Chicago, Ill, USA).

Differences for continuous variables between the two groups were analyzed by the student’s t-test or Mann-Whitney U test according to spread of data. Chi-square test was performed for categorical variables. The Pearson or Spearman correlation test was used to define the correlations between thyroid function test values and gestational age, as appropriate. Friedman test was used for repeated-measures analysis. Variables are given as mean±standard deviation values unless otherwise indicated. A p-value of less than 0.05 was accepted as significant.

## RESULTS

At final evaluation, it was found that RDS (n=56, 37.8%), ICH (n=34, 23%), NEC (n=29, 19.6%), PDA (n=12, 8.1%), BPD (n=6, 4.7%), and ROP (n=7, 4.7%) were among the most frequently diagnosed conditions in the studied infants. Sepsis developed in 47 subjects (31.7%) with 84 episodes during the study period. Clinical and proven sepsis rates were 7.4% (n=11) and 8.1% (n=12), 15.5% (n=23) and 12.2% (n=18), 8.8% (n=13) and 4.7% (n=7) at 1st, 2nd and 4th weeks, respectively. Maternal, perinatal and postnatal characteristics of the subjects according to T3 groups are presented in Table 1.

T3 values were lower than 1 nmol/L in 22 (14.9%), 31 (20.9%), and 10 (6.8%) infants at the 1st, 2nd, and 4th weeks, respectively. At the first evaluation, gestational age and Apgar scores were lower, but RDS rate was higher in the low T3 group. At the 4th week, mortality rate was higher in the first group. Sepsis was more frequent in the low T3 group during all study periods. IL-6 and CRP values were significantly higher during the sepsis attacks (Table 2).

Table 3 shows the results of thyroid function tests as well as the serum levels of IL-6 and CRP at the 1st, 2nd, and 4th weeks of life. IL-6 and CRP values significantly increased from the 1st week to the 2nd week and decreased thereafter. T3 values increased from the 1st week to the 4th week, although not significantly. T4 values significantly increased during the study period. No significant change was observed in fT3, fT4 and TSH values over the 1st to 4th week period. Table 4 shows the relation of high IL-6 and CRP values with low T3 values during the study period. The proportion of infants with high IL-6 or CRP was significantly higher in the low T3 group during all three evaluation periods.

**Figure 1:**
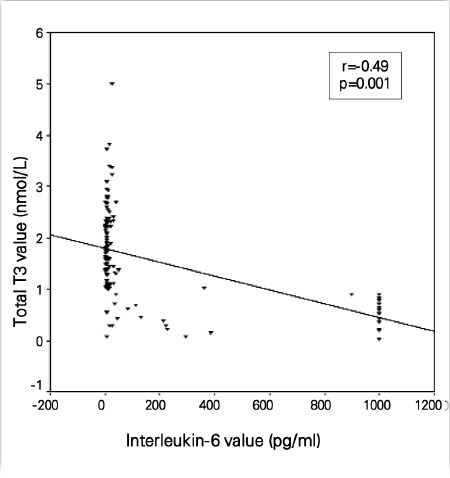
Scatter plot graph of correlation between interleukin-6 and totaltriiodothyronine (T3) value (nmol/L) at 2^nd^ week of life

**Figure 2:**
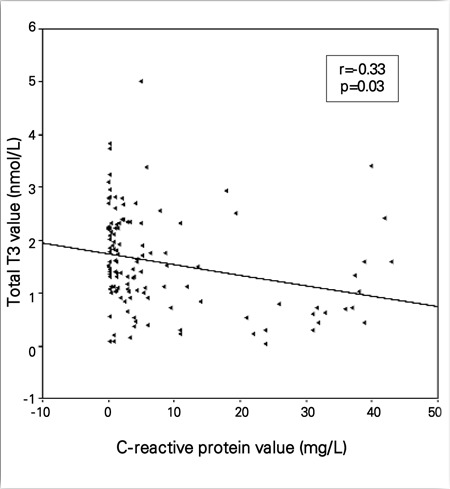
Scatter plot graph of correlation between C-reactive protein andtotal triiodothyronine (T3) value (nmol/L) at 2^nd^ week of life

When all subjects were analyzed together, serum T3 was negatively correlated with IL-6 (r=-0.33, p=0.001) and CRP (r=-0.17, p=0.03). fT3, fT4, T4, and TSH did not correlate with IL-6 and CRP at the 1st week. There was also a significant correlation between IL-6 and CRP (r=0.001, p=0.31). At the 2nd week, the period at which the frequency of sepsis and low T3 rate were the highest, serum T3 level was negatively correlated with IL-6 (r=-0.49, p=0.001) and CRP (r=-0.33, p=0.03) levels (Figures 1 and 2). fT3 was negatively correlated with CRP (r=-0.22, p=0.01) but not with IL-6, fT4, and T4; TSH did not correlate with IL-6 and CRP.There was a significant correlation between IL-6 and CRP (r=0.001, p=0.49). At the 4th week, serum T3 was negatively correlated with 

CRP (r=-0.25, p=0.02). There was a correlation between IL-6 and T3 although not statistically significant(r=-0,13, p=0.22). fT3 was negatively correlated with CRP(r=-0.22, p=0.01), but not with IL-6, fT4, T4; TSH did not correlate with IL-6 and CRP. There was a significant correlation between IL-6 and CRP (r=0.001, p=0.53).

## DISCUSSION

Abnormalities in the hypothalamus-pituitary-thyroid axis have recently been shown to be prevalent in newborns and children (19). Das and coworkers (20) suggested that low total T3 and T4 levels were predictors of adverse outcome in neonates with sepsis. In 2004, Yildizdas and coworkers (21) measured thyroid hormone levels and investigated their relationship with survival in children with bacterial sepsis and septic shock. In a similar study, den Brinker and coworkers (22) showed that all children surviving meningococcal septic shock had signs of NTI on admission. Based on these changes in the hypothalamus-pituitary-thyroidal axis, it can be speculated that thyroid hormone levels may have a possible prognostic value in children with sepsis and septic shock. In this study, we observed that while sepsis rate decreased, T4 values increased over the 1st week to the 4^th^ postnatal week.

Inflammatory cytokines such as IL-1 and IL-6 have been implicated in the pathogenesis of NTI (10). In a study of 20 normal volunteers, administration of IL-6 was followed by a decrease in serum TSH concentration, between 3 and 4 hours post injection. TSH levels returned to normal by 24 hours. Serum T3 and rT3 levels did not change acutely; T3 concentration decreased and rT3 increased at 24 hours (23). In another study, Eggum et al (24) reported a positive correlation between IL-6 values and serum concentrations of T3 in children during cardio-pulmonary bypass. In this present study, serum fT3 levels were found to be negatively correlated with IL-6 values at the 2^nd^ week of the study, a time at which the sepsis rate was the highest. In another study, Boelen et al (25) reported that cytokines had no role in NTI. In adult patients with chronic kidney disease, Abozenah et al (16) observed substantially high levels of IL-6 in patients with NTI, supporting its possible role as an endocrine cytokine with a regulatory effect on many endocrine systems including the thyroid gland. The high IL-6 and low T3 serum levels in sick premature infants might be independent expressions of the acute phase response during illness, and a still unknown component of the acute phase response may have been responsible for generation of NTI. In the present study, we showed that serum IL-6 levels correlated with T3 levels.CRP is predominantly induced by cytokines, especially by IL-6, and is produced in the liver, whereas iodothyronine5'-deiodinase, mediating the conversion of T3 from T4, is also found in rich quantities in the liver. IL-6 suppresses the secretion and gene expression of thyroxine-binding globulin in the Hep G2 hepatoblastoma-derived cell line. It is possible that IL-6 both stimulates the production of CRP and inhibits iodothyronine 5'-deiodinase activity (15). Although some previous studies have indicated that inflammatory cytokines, including IL-6, were released in uninfected infants with perinatal complications, the results obtained to date remain discordant (26). In recent years, based on the premise that their increase in response to infection may precede that of CRP, the search for diagnostic tests for early-onset sepsis in newborns has led to a consideration of cytokines, alone or in combination with CRP (27). Hashimoto et al (15) showed that serum T3 concentrations in patients with an increased IL-6 level were significantly reduced during acute infections in children. In our study, it was observed that the relation between IL-6 and T3 was more striking at the 2^nd^ week of life, the time at which sepsis rate was also the highest. Interestingly, only CRP was significantly correlated with T3 at the 4^th^ week of life. It can be speculated that CRP is more suitable than IL-6 to evaluate the relation of T3 with sepsis at the chronic phase of the infection.There is no consensus in the literature regarding the diagnosis and therapy of NTI. It can be said that the pathogenesis of NTI is multifactorial. Animal studies reveal no clear evidence on the positive or negative effect of T4 or T3 treatment in NTI (28). Because of the underlying disorder, these patients will not be “cured” just by adding thyroid hormones to the treatment. However, T4 or T3 may be a part of the list of multiple drugs which NTI patients receive to reduce their morbidity and mortality.

In summary, we have demonstrated that there exists a significant negative correlation between serum IL-6 and CRP concentrations and circulating thyroid hormone concentrations in premature infants with NTI. The observed increase in T4 levels from the 1st to the 4^th^ week of life may be related to decrease in sepsis rate as well as to increased postnatal age. Further studies are needed to explain the role of acute phase reactants in NTI in premature infants.

## Figures and Tables

**Table 1:**
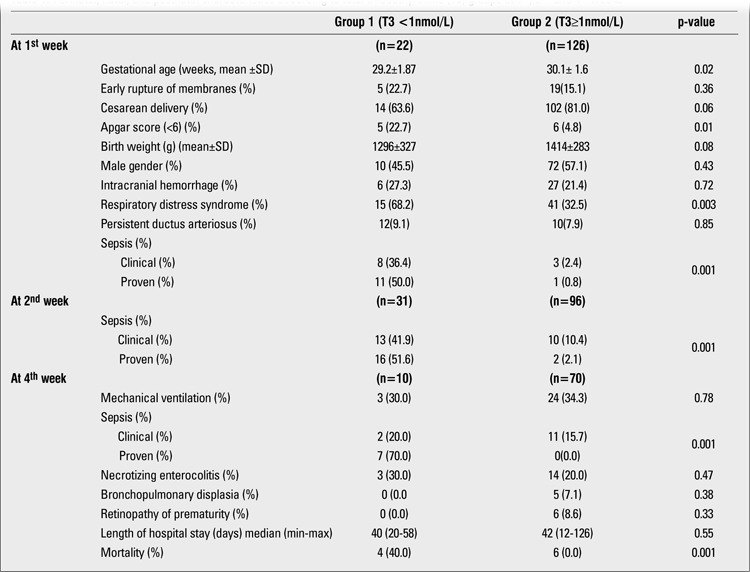
Perinatal, natal, and postnatal characteristics according to total triiodothyronine (T3) groups at 1^st^, 2^nd^ and 4^th^ weeks

**Table 2:**
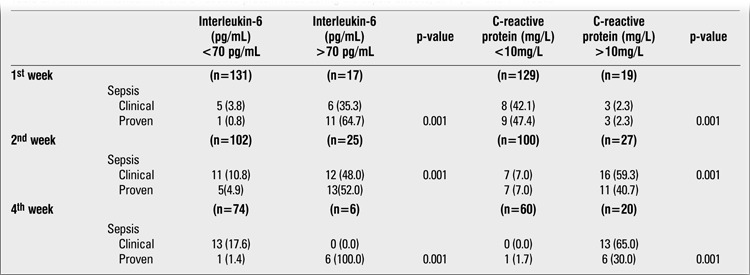
Abnormal interleukin-6 and C-reactive protein rates during the sepsis attacks, at 1^st^, 2^nd^ and 4^th^ weeks

**Table 3:**
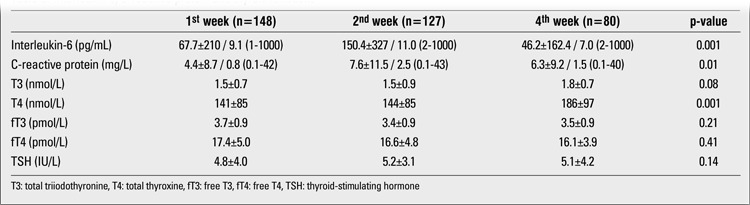
Interleukin-6, C-reactive protein and thyroid functions

**Table 4:**
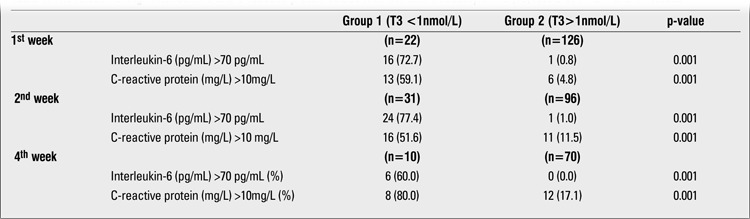
The relation of high interleukin-6 and C-reactive protein values with low total triiodothyronine (T3) levels at 1^st^, 2^nd^ and 4^th^ weeks
